# The Development of INT131 as a Selective PPAR*γ* Modulator: Approach to a Safer Insulin Sensitizer

**DOI:** 10.1155/2008/936906

**Published:** 2008-08-26

**Authors:** Linda S. Higgins, Christos S. Mantzoros

**Affiliations:** ^1^InteKrin Therapeutics, Inc., 4300 El Camino Real, Suite 201, Los Altos, CA 94022, USA; ^2^Division of Endocrinology, Diabetes and Metabolism, Beth Israel Deaconess Medical Center, Harvard Medical School, Boston, MA 02215, USA

## Abstract

INT131 (formerly T0903131, T131, AMG131) is a potent non-thiazolidinedione (TZD) selective peroxisome proliferator-activated receptor *γ* modulator (SPPARM) currently in Phase 2 clinical trials for treatment of type-2 diabetes mellitus (T2DM). This new chemical entity represents a second generation SPPARM approach developed after the first generation PPAR*γ* full agonists to address their inherent limitations. INT131 was specifically and carefully designed using preclinical models to exhibit a biological profile of strong efficacy with *de minimis* side effects compared to PPAR*γ* full agonists. As a potent PPAR*γ* modulator, INT131 binds to PPAR*γ* with high affinity. In pharmacology models of diabetes and in early clinical studies, it achieved a high level of efficacy in terms of antidiabetic actions such as insulin sensitization and glucose and insulin lowering, but had little activity in terms of other, undesired, effects associated with TZD PPAR*γ* full agonists such as edema and adipogenesis. Ongoing clinical development is directed at translating these findings into establishing a novel and effective treatment for T2DM patients with an improved safety profile in relation to that currently available.

## 1. PPAR*γ* FULL AGONISTS

PPAR*γ* full agonists are a mainstay in the treatment of insulin resistance and type-2 diabetes. While the glucose lowering action of thiazolidinediones (TZDs) was well-known as early as 1988 [1], it was not until 1995 that the nuclear receptor PPAR*γ* was identified as their target [2] and that its activation was shown to be responsible for their therapeutic benefits. PPAR*γ* full agonists, including the TZDs rosiglitazone (Avandia) and pioglitazone (Actos) are powerful drugs for the treatment of insulin resistance associated with type-2 diabetes mellitus (T2DM) [3]. Troglitazone (Rezulin) was the first TZD approved for clinical use in the US in 1997, but was subsequently withdrawn from the market in 2000 due to idiosyncratic hepatotoxicity. Rosiglitazone and pioglitazone were approved in the US in 1999. These drugs enabled the beneficial effect of PPAR*γ* activating agents to be recognized in clinical practice globally.

These medications enhance insulin sensitivity and reduce glucose and insulin levels in T2DM patients, and have been shown to have robust and relatively durable benefit for glucose control [4]. Insulin resistance is a key etiologic feature in the onset and subsequent progression of the disease. Furthermore, insulin sensitization comprises a complementary mechanism of action to that of other commonly used therapeutic modalities such as inhibition of gluconeogenesis by metformin, increased insulin secretion by sulfonylureas, and administration of exogenous insulin. The potential to be used in combination with other approaches thus further extends the clinical utility of PPAR*γ* activating agents for glucose control and to treat T2DM. Rosiglitazone and pioglitazone, both in the TZD class, are the only agents currently approved for insulin sensitization as their major mechanism of action.

Realization of PPAR*γ* maximal therapeutic potential by full agonists is limited, however, by associated side effects. PPAR*γ* full agonist binding to PPAR*γ* activates a broad spectrum of PPAR*γ* mediated effects, some of which are undesirable. Thus, use of TZDs is limited by side effects that include weight gain, fluid retention, and decreased bone density [5]. TZD-induced peripheral edema, which frequently occurs in patients receiving TZD monotherapy, is especially problematic in patients receiving concomitant insulin therapy, and is of special concern for patients who have either clinical or subclinical congestive heart failure (CHF) and thus cannot tolerate the extra fluid volume [6, 7]. In addition, there is strong evidence that activation of PPAR*γ* causes adipocyte differentiation and increased adipose tissue mass, contributing to weight gain [3]. The dose response curve for the therapeutic effects of TZDs overlaps with the dose response for side effects, such that increasing doses produce both greater benefits for glucose control as well as greater incidence and higher degrees of side effects [8]. Thus, doses which would produce the maximal clinical benefit of PPAR*γ* full agonists may not be tolerated by a significant number of patients and the full potential of PPAR*γ* activation for insulin sensitization and glucose control may not be realized at approved clinical doses of rosiglitazone or pioglitazone.

As a consequence of the known safety issues, TZDs are not recommended for patients with New York Heart Association Class 3 and 4 CHF, and the potential clinical impact of cardiovascular side effects prompted the American Heart and the American Diabetes Associations to issue a joint consensus statement advising against the use of TZDs in patients with advanced heart failure [9]. Awareness of the safety issues associated with TZDs was dramatically increased following the publication of a meta-analysis in May of 2007 showing a nonstatistically significant trend towards an increase in macrovascular events in patients taking rosiglitazone [10]. As a result of a detailed examination of the safety record for the TZD class, both rosiglitazone and pioglitazone received black box safety warnings for the increased risk of CHF due to fluid retention. Only rosiglitazone was further implicated for a “possible” risk of increased ischemic cardiovascular events [11] and obtained an additional black box warning, but data suggesting this risk have not been replicated by all studies. Finally, a series of scientific papers has demonstrated an association between TZD use and bone fracture, especially in women [12]. Despite these well-known limitations, Actos and Avandia represent a combined annual global market of more than $5 billion even following a rapid decrease and then stabilization of total sales and a switch from rosiglitazone to pioglitazone or other antidiabetic medications following heightened awareness of safety concerns in 2007. The continued use of the TZDs is a strong testament to the utility of insulin sensitization as a mode of action for treatment of T2DM, but also underscores the need for a safer treatment for insulin resistance.

**Figure 1:**
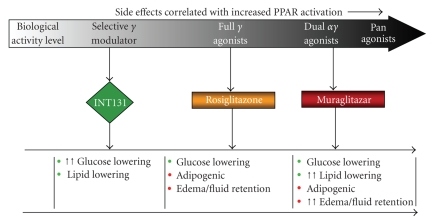
*Spectrum of PPAR*γ* effects*. The range of biological activities, both desired antidiabetic therapeutic effects and undesired effects related to tolerability and safety issues, increases for ligands characterized as antagonists, selective agonists, full agonists, and broad selectivity full agonists.

Historically, the proven therapeutic utility of activating the PPAR*γ* nuclear receptor to reduce glucose and HbA1c led the pharmaceutical industry to focus on a search for greater and broader efficacy through more potent PPAR*γ* full agonists as well as through the development of dual *α* and *γ* (“*α*/*γ*”) PPAR agonists. The latter were intended to combine the insulin sensitizing effects of PPAR*γ* activation with the lipid lowering effects of PPAR*α* activation. Unfortunately, no new agents deriving from these programs have been approved for clinical use. In the case of full PPAR*γ* agonists, efficacy and side effects have been shown to be intrinsically linked, with higher efficacy compounds associated with greater propensity for side effects. Similarly, PPAR*α*/*γ* dual agonists have been plagued with side effects. For example, muraglitazar, a dual PPAR*α*/*γ* agonist, was taken through a comprehensive development program and demonstrated remarkable efficacy in lowering HbA1c as well as improving lipid profile in T2DM patients. However, preclinical and clinical safety signals associated with edema, weight gain, and increased cardiovascular events led to a request in 2005 by FDA for outcome studies prior to approval and resulted in abandonment of the program by the sponsor in 2006. In summary, accumulated experience with PPAR*γ* and PPAR*α*/*γ* ligands has led to an understanding of a spectrum of desirable and undesirable activities, as graphically depicted in Figure 1.

## 2. SELECTIVE PPAR*γ* MODULATION SEPARATES EFFICACY AND SIDE EFFECT DOSE REPONSE CURVES

A very different approach to leveraging PPAR*γ* antidiabetic therapeutic benefits would focus on minimizing side effects (Figure 1, left) by limiting the spectrum of activation. This approach would require selective PPAR*γ* modulation which by design would minimize side effects while maintaining desired therapeutic benefit.

**Figure 2:**
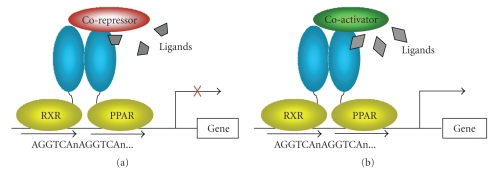
*PPAR*γ* activation*. Upon ligand binding, the nuclear receptor PPAR***γ*** associates with nuclear receptor RXR as well as with coactivators and corepressors which are present in a cell type and state specific pattern. This complex binds to PPAR response elements to enhance or repress gene transcription.

After the identification of PPAR*γ* as the target for TZDs, the crystal structure of the PPAR*γ* binding pocket as well as its activity relationships were probed, providing an important tool for pursuing selective modulation of the receptor. For example, in the case of the TZD PPAR*γ* full agonists, a key interaction occurs between the ligand and the activation helix (helix 12) of PPAR*γ* [13, 14]. Binding of activating ligands to the nuclear receptor PPAR*γ* leads to conformational changes favoring binding of PPAR*γ* to the RXR nuclear receptor, which is required for PPAR*γ* driven gene transcription, as well as to altered association with cofactors (Figure 2). Different types of PPAR*γ* ligands lead to sufficiently different conformations of the bound receptor heterodimer complex that different combinations and patterns of coactivators and corepressors are recruited for differential transcriptional control [15]. That is, the composition of the protein complex of PPAR*γ*, RXR, and specific cofactors determines the pattern of the ensuing gene transcription and hence the cellular response to the PPAR*γ* ligand. Since the repertoire of cofactors available for recruitment to the PPAR*γ*-RXR complex varies among cell types, PPAR*γ* responses are context-dependent. Thus, full agonists such as TZDs would be expected to lead to a different pattern of cofactor recruitment, gene transcription, and cellular response than a SPPARM.

**Figure 3:**
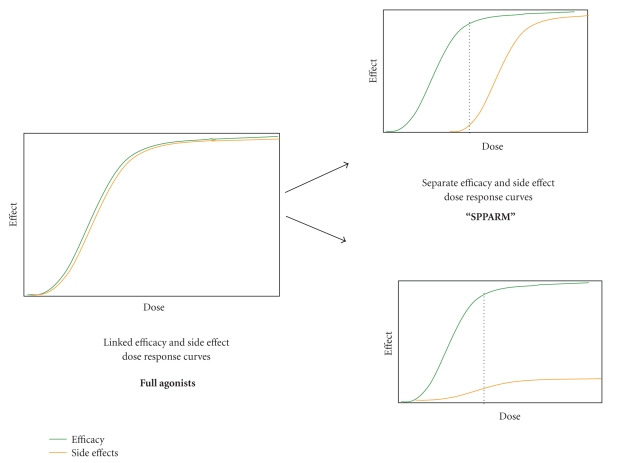
*Selective PPAR*γ* modulation separates dose response curves of different PPAR*γ* effects*. *Left*: PPAR*γ* full agonists activate the range of receptor responses in a linked fashion. Hence, increasing concentration (or dose) increases responses in concert. In the clinical setting, higher doses of TZDs produce greater efficacy as well as greater side effects. *Right*: selective PPAR modulation is response and context-dependent. Depending on the cellular setting and the response being measured, SPPARM activity may have different potency (top) or different maximal activity (efficacy, bottom) compared to a full agonist. Hence, increasing concentration (or dose) may lead to increases in some responses without linked increases in others. This offers the potential in the clinical setting for separation of antidiabetic efficacy from side effects such as edema and weight gain.

Theoretically, SPPARMs can be identified or designed which would produce a pattern of cofactor recruitment, gene transcription, and cellular response whereby the dose response curves for desired and undesired effects seen in patients could potentially be sufficiently separated to establish a broad therapeutic window (Figure 3). Is there precedence for the success of a modulator approach for another nuclear receptor? Both tamoxifen and its successor raloxifene are selective estrogen receptor modulators (SERMs) which are designed to optimize the therapeutic actions of estrogen receptor activation while minimizing the side effects [16]. A number of SPPARMs have to date been identified by in vitro and preclinical studies and some have entered early clinical studies [11, 14] but no reports have been published on any of these molecules reaching advanced stages of clinical development.

## 3. NT131 SPECIFIC DESIGN AND DEVELOPMENT FOR MOLECULAR AND IN VITRO SPPARM ACTIVITY

 INT131 (formerly T0903131, T131, AMG131) was developed focusing on a strategy to design a SPPARM which would bind to PPAR*γ* with high affinity but could potentially activate only a subset of the full spectrum of activities. Such a specifically designed molecule would thereby retain the antidiabetic actions of full PPAR*γ* agonists such as rosiglitazone and pioglitazone but would have minimal, if any, side effects (including weight gain and fluid retention) caused by these TZDs. In fact, a primary screening assay assed only moieties which *antagonized* roziglitazone induced activity associated with side effects INT131 was thus designed and developed as a non-TZD PPAR*γ* modulator which represents a new chemical class of PPAR*γ* ligands. INT131 binds to PPAR*γ* within the same binding pocket as the TZDs, but occupies a unique space in the pocket and contacts the receptor at distinct points from the TZDs [17]. Importantly, the interaction with the activation helix of PPAR*γ* by INT131 and by TZDs differs. The net result of the different binding by the two types of ligands is alternative conformational change of PPAR*γ*, leading to distinct patterns of association with cofactors by this nuclear receptor, and thus ultimately to unique patterns of gene transcription [15, 17].

**Figure 4:**
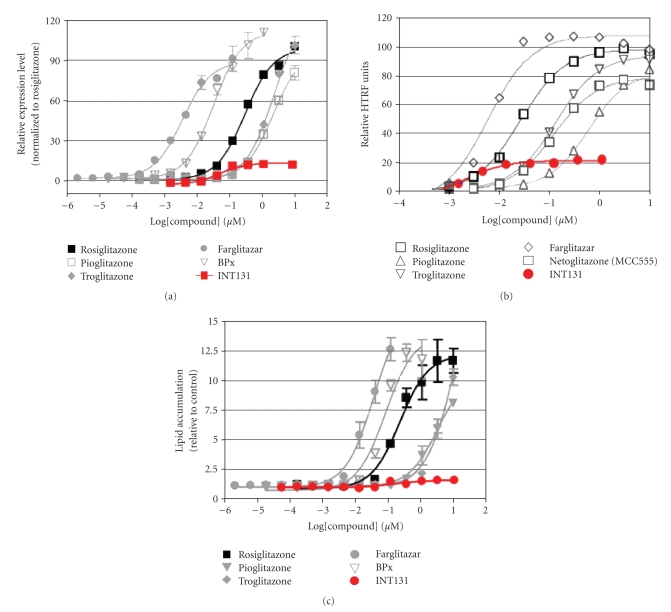
*PPAR*γ* full agonists, but not INT131, activate expression of a full agonist reporter gene, induce recruitment of DRIP205 coactivator peptide to PAPR*γ*, and cause lipid accumulation*. (a) An expression construct bearing a PPAR response element designed to be activated by PPAR*γ* full agonists was used to detect reporter gene expression. Transfected HEK cells were exposed to a range of concentrations of the indicated PPAR ligands, and expression measured. The maximal expression stimulated by INT131 was about 10% that promoted by rosiglitazone, pioglitazone, troglitazone, farglitazar, and BPx. (b) A homogenous time-resolved fluorescence energy transfer (FRET) assay was used to measure association of a DRIP205 coactivator peptide to PPAR*γ* upon exposure to a range of concentrations of the indicated PPAR ligands. The maximal association stimulated by INT131 was about 20–25% that was promoted by rosiglitazone, pioglitazone, troglitazone, farglitazar, and netoglitazone. (c) Lipid accumulation was measured in murine preadipocytes exposed to a range of concentrations of the indicated PPAR ligands. The maximal lipid accumulation stimulated by INT131 was about 10% that was promoted by rosiglitazone, pioglitazone, troglitazone, farglitazar, and BPx, Data on file.

 INT131 binds to PPAR*γ* and displaces rosiglitazone with a Ki of ~10 nM [17], demonstrating ~20-fold higher affinity than either rosiglitazone or pioglitazone [18], and with greater than 1000-fold selectivity for PPAR*γ* over PPAR*α*, PPAR*δ*, or a set of other nuclear receptors [17]. Characterization beyond binding reveals that selected PPAR*γ* receptor activities are induced by INT131. In a cell-based reporter assay designed to detect full agonist activity, INT131 activates PPAR*γ* with an efficacy of only about 10% of that of rosiglitazone (Figure 4(a)). Similarly, in fluorescence resonance energy transfer assays, INT131 causes recruitment of coactivator DRIP205, which is important for adipocyte differentiation, with an efficacy of about 20–25% of that of a set of full agonists including rosiglitazone, pioglitazone, and troglitazone (Figure 4(b)). Consistent with its high potency, selective activity profile in the full agonist cell-based reporter and FRET assays, INT131 causes little adipocyte differentiation or triglyceride accumulation in cultured mouse (Figure 4(c)) or human preadipocytes [17, 19]. Moreover, INT131 blocks most of the potent effects of rosiglitazone to promote fat cell differentiation [17]. Thus, INT131 shows selectivity among the full spectrum of PPAR*γ* effects and has the desired, nonadipogenic profile.

**Figure 5:**
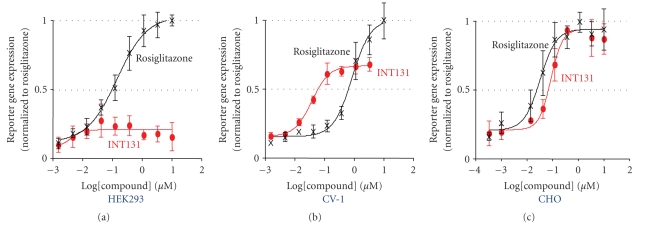
*PPAR*γ* activation by INT131 is cell-type-dependent.* Cell-based reporter assays were performed by transfecting three different cell types (HEK293, CV-1, CHO) with the same reporter construct and stimulating with increasing concentrations of rosiglitazone (black) or INT131 (red). Adapted from [17].

PPAR*γ* activation by a SPPARM is predicted to be context-dependent. Maximal activity of INT131 is sensitive to cellular environment of PPAR*γ*. That is, using the same reporter construct and assay designed to detect PPAR*γ* full agonist activity, INT131 potency and efficacy may be less than, equal to, or greater than the comparator full agonists rosiglitazone depending on the host-cell type (Figure 5).

## 4. PHARMACOLOGY OF INT131 IS CONSISTENT WITH SPPARM ACTIVITY

**Figure 6:**
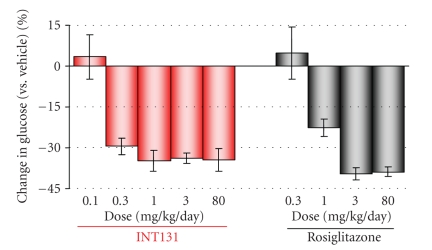
*Glucose level in Zuker fatty rat is reduced in response to either INT131 or rosiglitazone treatment.* Fourteen-day treatment with the indicated daily oral dose of INT131 or rosiglitazone in increasing doses reduce glucose levels. Adapted from [17, 19].

**Figure 7:**
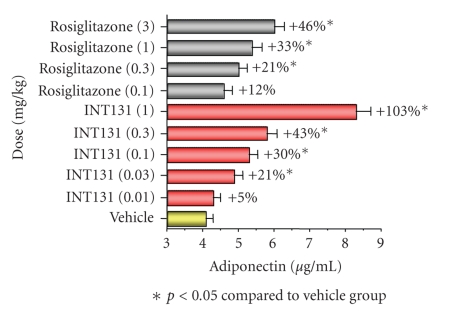
*Adiponectin levels increase in a dose responsive manner following either INT131 or rosiglitazone treatment*. Zucker fatty rats were treated orally with the indicated dose of INT131 or rosiglitazone once daily for 15 days, and plasma adiponectin was measured. *P* < 0.05 compared to vehicle group adapted from [17, 19].

INT131 is potent and highly efficacious in animal models of diabetes, but causes much less weight gain and volume expansion than marketed TZDs. For example, in Zucker fatty rats, a standard rodent model of T2DM, INT131 was more potent than rosiglitazone in reducing serum glucose (Figure 6), insulin, triglyceride, and NEFA concentrations and in improving glucose tolerance [17]. Notably, INT131 increased levels of the adipokine adiponectin in the Zucker fatty rat model and in normal rats with equal or greater potency than does rosiglitazone (Figure 7). Adiponectin levels are suppressed in obesity and in T2DM, and increased adiponectin production is thought to be a key mediator for the insulin sensitizing and anti-inflammatory effects of PPAR*γ* [20].

**Figure 8:**
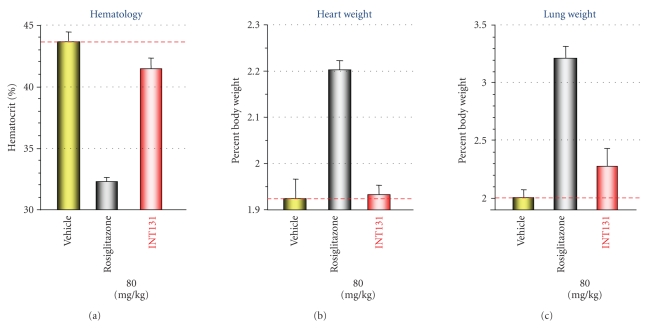
*INT131 does not increase plasma volume, heart weight, or lung weight.* Zucker fatty rats were treated orally once daily for 14 days with 80 mg/kg/day of INT131 or rosiglitazone (*n* = 6/group). (a) Hematocrit, (b) heart weight, and (c) lung weight were measured, and organ weights normalized to body weight. Adapted from [17, 19].

In a variety of animal models, full agonists cause fluid retention and increased heart weight, probably as a result of the increased cardiac load caused by plasma volume expansion. As expected, administration of rosiglitazone to Zucker diabetic fatty rats for two weeks caused a significant decrease in hematocrit, a marker for increased plasma volume expansion (Figure 8(a)); increase in heart weight (Figure 8(b)); and increased lung weight (Figure 8(c)) consistent with a secondary effect to cardiac hypertrophy and developing CHF. INT131 at the same supratherapeutic dose did not cause these effects. Thus, SPPARM activity is observed in this rodent model of T2DM, and the antidiabetic effects of PPAR activation have been separated from fluid retention and adverse cardiac effects.

## 5. TOXICOLOGY OF INT131 DEMONSTRATES A SAFETY PROFILE DISTINCT FROM TZDs AND CONSISTENTWITH A SPPARM

**Figure 9:**
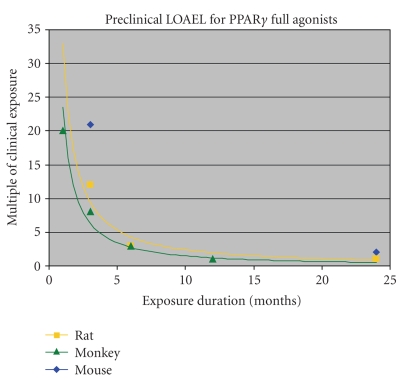
*Preclinical LOAEL for PPAR*γ* full agonists follows a steep time dependence.* The lowest observable adverse effect level (LOAEL) at various exposure times in mouse, rat, and monkey is depicted for aggregate data for the class and expressed as multiple of clinical exposure level. Data from [21].

 Preclinical safety experience with PPAR*γ* full agonists has produced a consistent profile of target mediated effects. Prominent among these are: fluid retention as manifested by a drop in hematocrit and related hematological measures of increased plasma volume as well as in edema; weight gain due to increased adipose tissue together with fluid retention; cardiac hypertrophy and heart failure; and fatty infiltration and replacement of bone marrow. Appearance of these adverse effects follows a predictable steep time and dose relationship in multiple species (Figure 9, [21]), and has been predictive of clinical experience. Therefore, preclinical results from subchronic and chronic safety studies take on heightened importance for PPAR ligands in clinical development. Based on experience with many PPAR full agonist programs, the 2008 FDA draft guidance for development of diabetes drugs [22] includes specific recommendations for preclinical studies with PPAR ligands. These include detailed measures to detect cardiac changes, fatty infiltration of organs, and fluid retention. According to the draft guidance, appearance of safety signals in preclinical programs which have been predictive of clinical safety issues for other PPAR ligands could lead to a requirement for more detailed clinical safety studies or outcome studies prior to approval.

INT131 is well tolerated in rats treated for 6 months with doses resulting in up to two to three orders of magnitude greater exposure than exposure attained at efficacious clinical doses in humans. Of particular note was the lack of the toxicities characteristic of PPAR*γ* full agonists, including signs of fluid accumulation or increased heart weight at doses representing these high safety multiples. These adverse effects are typically observed at or near efficacious exposure levels for potent PPAR*γ* full agonists. Thus, the therapeutic window for INT131 is predicted to be significantly greater than it is for the older classes of compounds.

Safety testing of INT131 in cynomolgus monkeys for one and six months at exposures up to >70-fold (highest dose and duration tested) over the exposures expected at the highest dose in the ongoing clinical development program showed that all doses were well tolerated. Confirming the rat safety study results, typical PPAR full agonist effects such as fluid retention, increased adiposity, fatty replacement of marrow, or cardiac changes detected by echocardiography, pathology, or histology were not observed in INT131 treated monkeys.

An additional area of concern for the general PPAR ligand class of compounds is carcinogenicity. In July 2004, FDA provided guidance regarding preclinical and clinical safety assessments for any molecules in clinical development affecting PPAR superfamily members. Cumulative rodent data reviewed by the agency for a number of PPAR*γ* dual *α*/*γ* agonists in development had shown an increased incidence of carcinogenicity. Based on these data, the FDA mandated that clinical dosing could not exceed six months with any PPAR ligand (*α*, *γ*, *δ*, *α*/*γ* dual, or *α*/*γ*/*δ* pan agonist) unless two-year rodent carcinogenicity studies were completed and satisfactorily reviewed by the agency.

**Figure 10:**
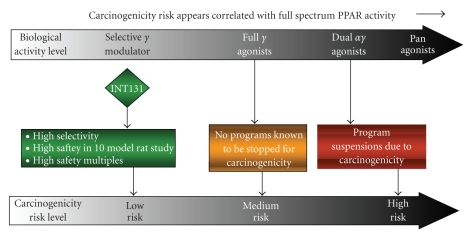
*Carcinogenicity risk in relation to spectrum of PPAR activation.* Propensity for demonstrating carcinogenic activity is predicted to increase with broader PPAR activating activity.

SPPARMs such as INT131 would appear to be at lower risk for demonstrating carcinogenic activity than PPAR*γ* full agonists and dual PPAR*α*/*γ* agonists (Figure 10) for several reasons. First, many of the PPAR binding molecules that caused tumors in the rodent studies were PPAR*α*/*γ* dual agonist with which multispecies, multitissue, and both-sex tumor incidence occurred [23]. INT131 is highly selective for PPAR*γ*, with no binding to PPAR*α* or *δ* at 10 *μ*M, 1000 fold over the Ki for PPAR*γ* [19].

While carcinogenicity is less of a concern for PPAR*γ* agonists than for PPAR*α* or *α*/*γ* dual agonists, the two most prevalent types of tumors associated with PPAR*γ* full agonist molecules which do occur are lipomas and hemangiosarcomas. These cancers derive from adipose tissue and vascular endothelium, respectively. Since INT131 shows little propensity to promote adipocyte differentiation in vitro or adipose proliferation in vivo, it would be reasonable to expect that INT131 would convey minimal, if any, risk for these malignancies. Similarly, the lack of edema in preclinical models suggests a weak activity in the vascular endothelium and thus would be unlikely to invoke the activation associated with hemangiosarcomas at very high doses of full PPAR*γ* agonists. Taken together, it is likely that selectivity of a SPPARM such as INT131 will reduce the potential for carcinogenicity that plague PPAR full agonists, but this remains to be conclusively shown by ongoing studies.

## 6. EARLY CLINICAL RESULTS WITH INT131 SHOW SEPARATION OF EFFICACY FROM SIDE EFFECTS

**Figure 11:**
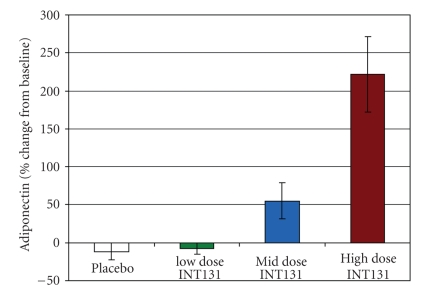
*
Adiponectin level is stimulated in human subjects exposed to INT131.* Circulating adiponectin was measured in healthy volunteers exposed to 8 consecutive daily fixed oral doses of INT131. Adapted from [24].

Four Phase 1 studies have demonstrated that INT131 besylate is well tolerated and has highly desirable pharmacokinetic and pharmacodynamic properties. The rapid and robust stimulation of adiponectin levels (Figure 11) provides evidence of activation of PPAR*γ* pathways associated with therapeutic efficacy, confirming preclinical pharmacology results [15].

A 4-week Phase 2a multicenter, randomized, double blind, placebo controlled study was conducted to establish the glucose lowering activity of INT131 besylate in subjects with T2DM. INT131 was well tolerated, with no significant safety signals [19]. A reduction in fasting plasma glucose (the primary endpoint of the study) was observed at week 1 and week 4, unusually early for this mechanism of action, and was statistically significant despite the short duration of treatment. Stimulation of adiponectin levels, seen in healthy volunteers in Phase I, was confirmed in the T2DM population in the Phase 2a study. Most notably, the SPPARM activity of INT131 was supported by separation of the observed antidiabetic effects from edema and weight gain, differentiating INT131 from TZD PPAR*γ* full agonists. These results provided the foundation for an ongoing multicenter double blind placebo controlled Phase 2b study of 4 doses of INT131 and pioglitazone comparator in T2DM patients, which is designed to rigorously test the SPPARM activity of INT131 for separation of PPAR*γ* mediated efficacy in treating insulin resistance from TZD side effects.

## 7. CONCLUSION

The non-TZD selective PPAR*γ* modulator INT131 is the culmination of a molecular target-based strategy to develop an improved insulin-sensitizing drug that does not cause the weight gain and edema that plague the PPAR full agonists. As predicted by its unique PPAR*γ* profile, INT131 shows potential as a potent and efficacious insulin-sensitizing molecule in T2DM patients that causes little if any weight gain at therapeutically efficacious doses. This emerging clinical profile of efficacy/side-effect separation is consistent with the underlying molecular biology design, the in vitro study data and the robust preclinical data. It thus represents the final part of an accordant continuum testing the hypothesis that selective modulation of PPAR*γ* can create a clinically relevant therapeutic window which is hoped to eventually provide tangible benefits to patients.

## References

[1] Fujiwara T, Yoshioka S, Yoshioka T, Ushiyama I, Horikoshi H (1988). Characterization of new oral antidiabetic agent CS-045. Studies in KK and ob/ob mice and 
Zucker fatty rats. *Diabetes*.

[2] Lehmann JM, Moore LB, Smith-Oliver TA, Wilkison WO, Willson TM, Kliewer SA (1995). An antidiabetic thiazolidinedione is a high affinity ligand for peroxisome 
proliferator-activated receptor *γ* (PPAR*γ*). *The Journal of Biological Chemistry*.

[3] Semple RK, Chatterjee VK, O'Rahilly S (2006). PPAR*γ* and human metabolic disease. *The Journal of Clinical Investigation*.

[4] Yki-Järvinen H (2004). Thiazolidinediones. *The New England Journal of Medicine*.

[5] Yki-Järvinen H (2005). The PROactive study: some answers, many questions. *The Lancet*.

[6] Mudaliar S, Chang AR, Henry RR (2003). Thiazolidinediones, peripheral edema, and type 2 diabetes: incidence, pathophysiology, 
and clinical implications. *Endocrine Practice*.

[7] Scheen AJ (2004). Combined thiazolidinedione-insulin therapy: should we be concerned about safety?. *Drug Safety*.

[8] Lago RM, Singh PP, Nesto RW (2007). Congestive heart failure and cardiovascular death in patients with prediabetes and type 2 diabetes given thiazolidinediones: a meta-analysis of randomised clinical trials. *The Lancet*.

[9] Nathan DM, Buse JB, Davidson MB (2008). Management of hyperglycemia in type 2 diabetes: a consensus algorithm for the initiation and adjustment of therapy: update regarding thiazolidinediones: a consensus statement from the American Diabetes Association and the European Association for the study of diabetes. *Diabetes Care*.

[10] Nissen SE, Wolski K (2007). Effect of rosiglitazone on the risk of myocardial infarction and death from cardiovascular causes. *The New England Journal of Medicine*.

[11] Goldfine AB (2008). The rough road for rosiglitazone. *Current Opinion in Endocrinology, Diabetes and Obesity*.

[12] Murphy CE, Rodgers PT (2007). Effects of thiazolidinediones on bone loss and fracture. *The Annals of Pharmacotherapy*.

[13] Nolte RT, Wisely GB, Westin S (1998). Ligand binding and co-activator assembly of the peroxisome proliferator- activated receptor-*γ*. *Nature*.

[14] Zhang F, Lavan BE, Gregoire FM (2007). Selective modulators of PPAR-*γ* activity: molecular aspects related to obesity and side-effects. *PPAR Research*.

[15] Olefsky JM (2000). Treatment of insulin resistance with peroxisome proliferator–activated receptor *γ* agonists. *The Journal of Clinical Investigation*.

[16] McDonnell DP (2003). Mining the complexities of the estrogen signaling pathways for novel therapeutics. *Endocrinology*.

[17] Li Y, Wang Z, Motani A T0903131 (T131): a selective modulator of PPAR*γ*.

[18] Willson TM, Brown PJ, Sternbach DD, Henke BR (2000). The PPARs: from orphan receptors to drug discovery. *Journal of Medicinal Chemistry*.

[19] McGee LR, Rubenstein SM, Houze JB Discovery of AMG131: a selective modulator of PPAR*γ*.

[20] Kadowaki T, Yamauchi T, Kubota N, Hara K, Ueki K, Tobe K (2006). Adiponectin and adiponectin receptors in insulin resistance, diabetes, and the metabolic syndrome. *The Journal of Clinical Investigation*.

[21] El Hage J Peroxisome proliferator-activated receptor agonists: preclinical and clinical cardiac safety 
considerations. http://www.fda.gov/ohrms/dockets/ac/05/slides/2005-4169S2_02_02-FDA-ElHage.ppt.

[22] US Department of Health and Human Services Food and drug Administration Center for Drug Evaluation and 
Research Guidance for Industry. Diabetes Mellitus: Developing Drugs and Therapeutic Biologics for Treatment and 
Prevention. http://www.fda.gov/OHRMS/DOCKETS/98fr/FDA-2008-D-0118-nad.pdf.

[23] El Hage J Preclinical and clinical safety assessments for PPAR agonists. http://www.fda.gov/CDER/present/DIA2004/Elhage.ppt.

[24] Kersey K, Floren LC, Pendleton B, Stempien MJ, Buchanan J, Dunn F T0903131, a selective modulator of PPAR-gamma activity, increases adiponectin levels in healthy subjects.

